# Evolution of Mortality by Age Group in Patients With COVID-19 Above 60 Years of Age

**DOI:** 10.7759/cureus.17149

**Published:** 2021-08-13

**Authors:** Maria Regina Pereira de Godoy, Maria Lucia Machaso Salomão, Flavia Queiroz, Nathalia Furquim Siqueira, Laura Fernandez Cardoso, Andrea Campos Indaló Saurin

**Affiliations:** 1 Clinica Medica, Faculdade de Medicina de Sao Jose do Rio Preto (FAMERP), Sao Jose do Rio Preto, BRA; 2 Epidemiologia e Saúde Coletiva, Faculdade de Medicina de Sao Jose do Rio Preto (FAMERP), Sao Jose do Rio Preto, BRA; 3 Epidemiologia e Saúde Coletiva, Hospital de Base, Faculdade de Medicina de São José do Rio Preto (FAMERP), Sao Jose do Rio Preto, BRA; 4 Serviço de Geriatria, Hospital de Base, Fundação Faculdade Regional de Medicina de São José do Rio Preto (FUNFARME), Sao Jose do Rio Preto, BRA

**Keywords:** mortality, covid-19, old, people, hospitalization

## Abstract

Introduction

The severity of the diseases associated with the coronavirus worsen the results, increasing the mortality of these patients.

Objective

The aim of the present study is to report coronavirus disease 2019 (COVID-19) hospitalizations and mortality in 2,359 patients aged over 60 years in a reference center in Brazil.

Method

The evolution of hospitalizations and overall mortality of patients admitted to Hospital de Base were evaluated in a clinical trial, then selecting patients aged over 60 years, by age group every 10 years, from March 2020 to April 2021, and analyzing whether there was an increase in mortality in age groups, assessed by Fisher's exact test.

Results

There was an increase in mortality over the decades in this period, but a reduction in hospitalizations after patient vaccination.

Conclusion

Mortality in patients over 60 years of age is higher than in younger patients, where the vaccine has reduced their hospitalization, but individual patient protection regarding mortality needs further evaluation.

## Introduction

Elderly people represent over 9% of the global population and over 6% of the Indian population. Coronavirus disease 2019 (COVID-19) has severely affected the elderly population where the identification of risk factors for serious illnesses and early intervention result in reduced mortality. The severity of diseases associated with the coronavirus worsen the results, increasing the mortality of these patients [1].

Chronic metabolic diseases are known risk factors for increased mortality after severe acute respiratory syndrome coronavirus 2 (SARS-CoV-2) infection. It is detected that there was a significant increase in the prevalence of obesity, dyslipidemia and metabolic syndrome compared to the pre-COVID-19 assessment [2].

Many lives ended prematurely and especially among older Americans, but the pandemic also had an indirect effect on non-COVID19 health and mortality among the working-age population, which was impacted by the economic consequences. Mortality rates increased during the two waves of COVID-19 then declined [3].

In the elderly, the disease progresses more quickly and complications develop more frequently. Other factors such as the severity of the SARS-CoV-2 pattern and the length of stay in the hospital in the final results of these patients [4]. The aim of the present study is to report the admissions and mortality of COVID-19 in people 2,359 patients over the age of 60 years in a reference center in Brazil

## Materials and methods

Casuistic and location

We evaluated 2,359 patients with positive COVID-19 in the age groups every 10 years, over 60 years, 1,055 women and 1,204 men, admitted to the Base Hospital of the Medicine School of São Jose do Rio Preto-SP-Brazil (FUNFARME), between March 2020 to April 2021.

Design

The evolution of hospitalizations and overall mortality of patients admitted to Base Hospital-Brazil were evaluated in a clinical trial, then selecting patients aged over 60 years, by age group every 10 years, from March 2020 to April 2021, and analyzing whether there was an increase in mortality in age groups, assessed by Fisher's exact test.

Inclusion criteria

All patients with COVID-19 admitted to the ward and intensive unit care (ICU) of the Base Hospital.

Exclusion criteria

Patients with deep venous thrombosis (DVT) and negative COVID-19 and treated on an outpatient basis.

Selection of patients

All patients with positive COVID-19 from March 2020 to April 2021 admitted to the ward and ICU of Base Hospital were included and then selected by age group over 60 years of age each decade.

Ethical consideration

The study was approved by the Research Ethics Committee # 4.802.791.

Development

The database related to the control of COVID-19 of Base Hospital was used, considering the number of hospitalized patients monthly, and overall mortality by age group was analyzed. Patients aged over 60 years, every 10 years, had their mortality assessed monthly. Data were included in an Excel table and evaluated by Fisher's exact test using the Stats Direct program.

Statistical consideration

Descriptive data statistics and Fisher's exact test were used, considering an alpha error of 5%.

## Results

**Figure 1:**
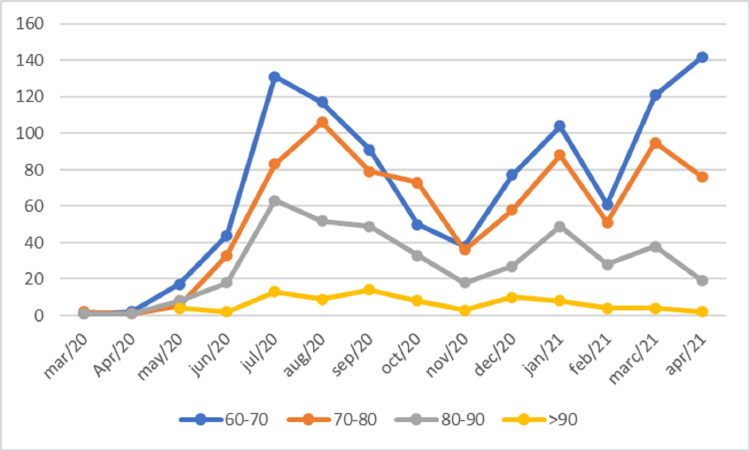
Evaluation of the number of patients hospitalized per month every 10 years between 60 and over years.

**Figure 2:**
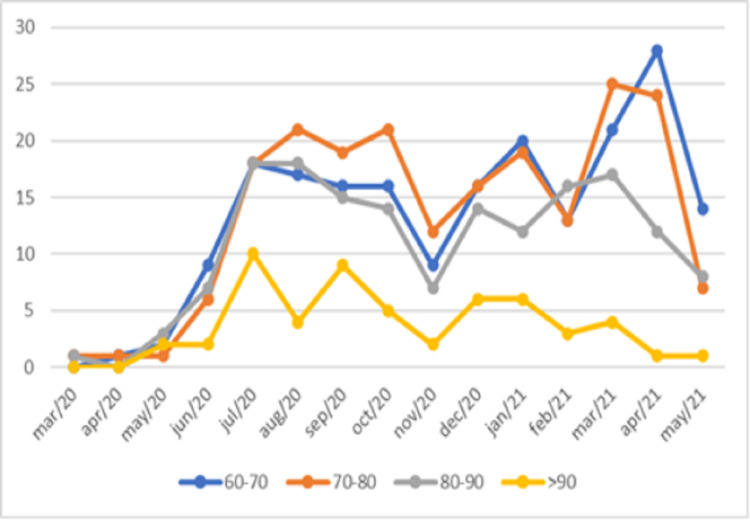
Variation of monthly mortality in age groups for decades after 60 years.

**Figure 3:**
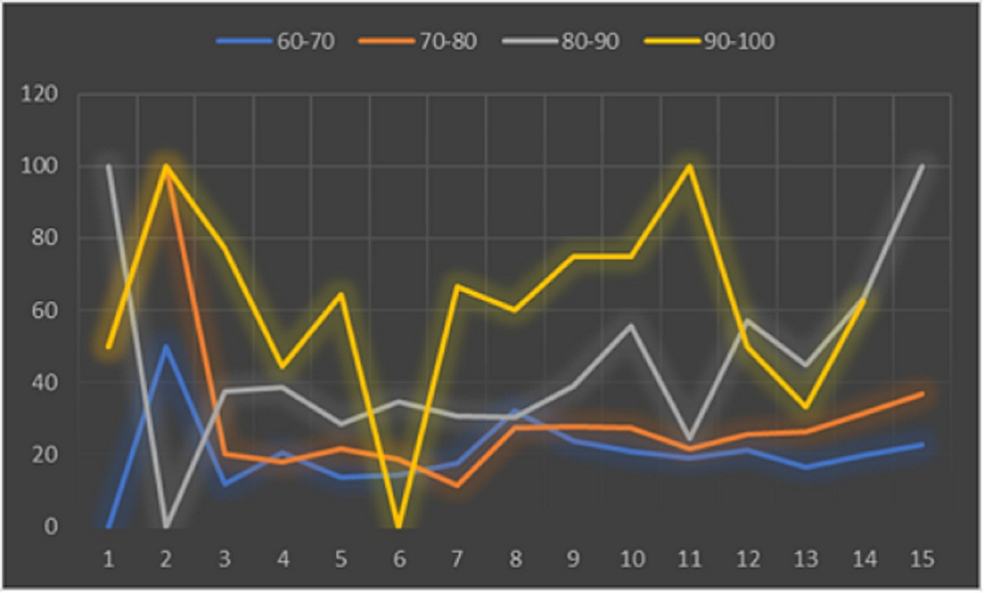
Monthly evolution of mortality percentage in the age groups every 10 years from 60 to over 90 years.

**Figure 4:**
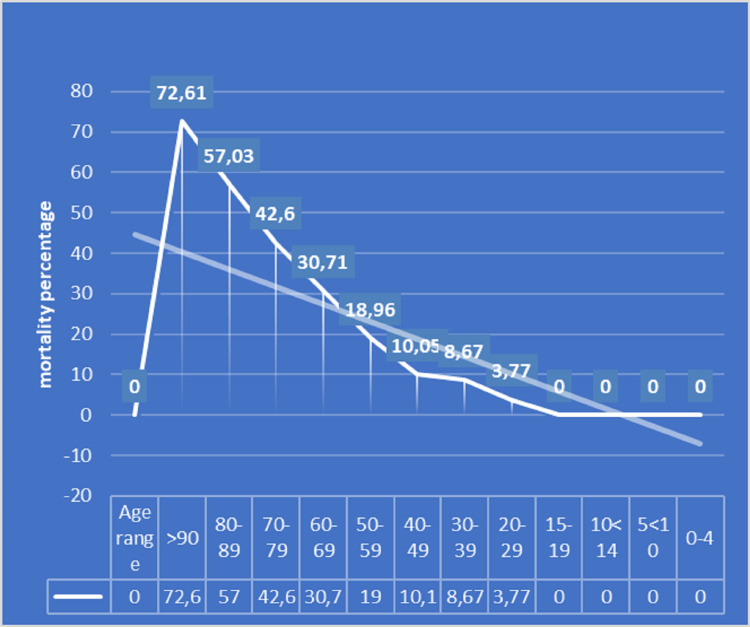
Monthly prevalence of overall mortality over the months by age group.

A total of 2,359 patients aged over 60 years were evaluated, varying by age group, as shown in Figure 1. Table 1 and Figure 2 show the monthly mortality by age group every 10 years. Table I shows the variation in monthly mortality in the age groups for decades after the age of 60 years and Figure 3 shows the monthly percentage evolution of mortality in the age groups. Figure 4 shows overall mortality over the months by age group. There is a significant increase in mortality in the age group over 60 years, Fisher's exact test p-value = 0.0001. There were 731 male patients with mortality of 143 patients and 664 women with 98 deaths. Men died more than women Fisher's exact test, p = 0.01.

**Table 1:** Variation of monthly mortality in age groups for decades after 60 years.

Age range	60-70	70-80	80-90	>90
Mar/20	1	2	1	
Apr/20	2	1	1	
May/20	17	5	8	4
Jun/20	44	33	18	2
Jul/20	131	83	63	13
Aug/20	117	106	52	9
Sep/20	91	79	49	14
Oct/20	50	73	33	8
Nov/20	38	36	18	3
Dec/20	77	58	27	10
Jan/21	104	88	49	8
Feb/21	61	51	28	4
Mar/21	121	95	38	4
Apr/21	142	76	19	2
Jun/21	62	41	19	3
Total	1.058	825	423	84

## Discussion

The present study reports the evolution of hospitalizations and monthly mortality in patients hospitalized with COVID-19, in the age groups every 10 years, above 60 years. In Figure 1, there is a reduction in intentions in the age groups above 70 years, while in the age group between 60 and 70 years there is an increase in the incidence. This age group had not been vaccinated while those over 70 years old had been, suggesting an interference in the prevention of covid with vaccines.

Another observation is that in the age groups from 40 to 80 years old, they had the highest number of hospitalized patients. Mortality increases with age, but after 60 years it is significantly higher in relation to younger age groups. It is believed that these data may be overestimated because almost all deaths in patients with COVID-19 were reported to be caused by COVID-19. Other causes of mortality were not always analyzed, increasing the current mortality related to COVID-19.

In the USA, they found that in the age groups below 15 years and above 65 years, the general mortality of the population was lower than the others. However, what we looked at was the in-hospital mortality associated with COVID-19, which increased with age. The association with other specific diseases is another aggravating factor reported throughout the literature and observed in these patients. They interfere in worldwide mortality and cardiovascular diseases, diabetes and obesity stand out.

One of the aggravations detected in the service was DVT which, when present, significantly increased patient mortality, a study in the process of being published. Early diagnosis and treatment are essential to reduce mortality and morbidity in these patients. Therefore, it is a set of measures that must be taken where prevention with vaccination of these patients is the main measure to be taken at the moment.

## Conclusions

Mortality in patients over 60 years of age is higher than in younger patients, where the vaccine has reduced their hospitalization, but individual patient protection regarding mortality needs further evaluation.

## References

[1] Klanidhi KB, Bhavesh M, Ranjan P, Chakrawarty A, Bhadouria SS (2021). Health care of the elderly during Covid-19 pandemic-All a family physician should know. J Family Med Prim Care.

[2] Auriemma RS, Pirchio R, Liccardi A, Scairati R, Del Vecchio G, Pivonello R, Colao A (2021). Metabolic syndrome in the era of COVID-19 outbreak: impact of lockdown on cardiometabolic health. J Endocrinol Invest.

[3] Glei DA (2021). The US midlife mortality crisis continues: increased death rates from causes other than covid-19. medRxiv.

[4] Dryha NO, Stepanenko AV, Rudenko LA, Zhaldak DO, Piven SM, Plakhtiienko IO (2021). Results of medical-social research on medical care quality for patients with covid-19 of inpatient hospital departments in Sumy region. Wiad Lek.

